# Addressing Doxorubicin Resistance in Bone Sarcomas Using Novel Drug-Resistant Models

**DOI:** 10.3390/ijms23126425

**Published:** 2022-06-08

**Authors:** Borja Gallego, Dzohara Murillo, Verónica Rey, Carmen Huergo, Óscar Estupiñán, Aida Rodríguez, Juan Tornín, René Rodríguez

**Affiliations:** 1 Instituto de Investigación Sanitaria del Principado de Asturias (ISPA), Hospital Universitario Central de Asturias, Avenida de Roma, s/n 33011 Oviedo, Spain; borjagallego@ispasturias.es (B.G.); dzomc@ispasturias.es (D.M.); veronicarey@ispasturias.es (V.R.); carmenhuergo@ispasturias.es (C.H.); ores@ispasturias.es (Ó.E.); aida.rodriguez@finba.es (A.R.); juantornin@ispasturias.es (J.T.); 2 Instituto Universitario de Oncología del Principado de Asturias, 33006 Oviedo, Spain; 3 CIBER en Oncología (CIBERONC), 28029 Madrid, Spain

**Keywords:** osteosarcoma, chondrosarcoma, doxorubicin, drug-resistance, cancer stem cells, patient-derived models

## Abstract

Bone sarcomas have not shown a significant improvement in survival for decades, due, in part, to the development of resistance to current systemic treatments, such as doxorubicin. To better understand those mechanisms mediating drug-resistance we generated three osteosarcoma and one chondrosarcoma cell lines with a stable doxorubicin-resistant phenotype, both in vitro and in vivo. These resistant strains include a pioneer model generated from a patient-derived chondrosarcoma line. The resistant phenotype was characterized by a weaker induction of apoptosis and DNA damage after doxorubicin treatment and a lower migratory capability. In addition, all resistant lines expressed higher levels of ABC pumps; meanwhile, no clear trends were found in the expression of anti-apoptotic and stem cell-related factors. Remarkably, upon the induction of resistance, the proliferation potential was reduced in osteosarcoma lines but enhanced in the chondrosarcoma model. The exposure of resistant lines to other anti-tumor drugs revealed an increased response to cisplatin and/or methotrexate in some models. Finally, the ability to retain the resistant phenotype in vivo was confirmed in an osteosarcoma model. Altogether, this work evidenced the co-existence of common and case-dependent phenotypic traits and mechanisms associated with the development of resistance to doxorubicin in bone sarcomas.

## 1. Introduction

Bone sarcomas comprise a rare group of mesenchymal tumors that represent less than 0.2% of cancer diagnoses [1]. Among this group of tumors, osteosarcomas (accounting for 35% of bone sarcoma diagnoses) and chondrosarcomas (25% of diagnoses) are the most common subtypes [1,2]. These sarcomas arise in the rich bone microenvironment in periods of increased bone formation and remodeling [3,4] and are characterized by highly complex karyotypes indicative of severe genetic and chromosomal instability [5,6,7]. Therapeutic options for bone sarcomas have remained largely unaltered for decades and usually involve the surgical resection of tumor mass, supported by radiotherapy or neoadjuvant chemotherapy [8,9]. In the case of advanced disease, or if surgery is not feasible, current treatments still rely on protocols using different cytotoxic drugs, and of these, doxorubicin (DOX), a topoisomerase II inhibitor that causes DNA damage-associated cell death, is the most widely used [8,9,10]. Despite their relatively low incidence, bone sarcomas represent a medical challenge, due to their aggressive behavior and their high rate of drug resistance to current treatments. Thus, more than 30% of patients with localized osteosarcoma, and more than 80% with metastatic/relapsed cancer, still succumb to the disease owing to inherent and/or acquired drug resistance [9,11,12]. In addition, drug resistance is a common feature of chondrosarcomas [13].

One of the most common phenotypic traits associated with the drug-resistant phenotype in osteosarcoma is the overexpression of efflux pumps of the ATP-binding cassette (ABC) family, such as ABCG2 (also known as BCRP) and ABCB1 (also known as MDR1 or P-glycoprotein), the expression of which is associated with a lower event-free survival rate in osteosarcoma patients [14,15,16]. Other well-known mechanisms of resistance to cytotoxic drugs include the expression of anti-apoptotic factors of the BCL-2 family [17,18], an increased DNA repair and detoxification ability or the adoption of a quiescent state [11,19,20,21]. All of these features are attributes of the so-called cancer stem cells (CSCs), which are subpopulations of tumor cells presenting stem cell properties that play key roles in tumor progression and are a source of drug resistance in bone sarcomas [22,23,24]. The acquisition of these pro-resistance phenotypes by osteosarcoma cells has been linked to the activation of signaling pathways, such as those controlled by WNT, NOTCH, hedgehog, HIF1, JAK/STAT3 or PI3K/mTOR, as well as to the expression of specific non-coding RNAs [11,19,23]. Based on this research, several pre-clinical studies have reported new treatments with potential to overcome drug resistance in bone sarcomas [9,19]. Also, the targeting of the tumor microenvironment with osteoclast inhibitors may result in a better response to chemotherapeutics and/or decreased metastatic spread [3,25]. Related to this, recent works have highlighted the potential role of denosumab, alone or in combination with tyrosine kinase inhibitors, as treatment for bone sarcomas [26,27,28]. In addition, the efficacy of the treatment of osteosarcoma and chondrosarcoma with the multiple kinase inhibitor Lenvatinib, in combination with the anti-PD-1 antibody pembrolizumab, or ifosfamide/etoposide, is currently being evaluated (NCT04784247) [29]. Moreover, other drugs able to target several pro-tumoral pathways at once have demonstrated potent anti-tumor activities in sarcomas alone or in combination with current treatments [30,31]. Among them, G-C rich DNA-binding compounds, such as mithramycin (MTM) and its analog EC-8042, have demonstrated a potent anti-stemness activity in sarcomas [32,33,34]. However, despite these advances in drug development, none of the alternative treatments tested so far in clinical trials have demonstrated clear advantages over DOX as first-line treatments [9]. The data highlights the need for intensified research to develop improved preclinical models to identify new synthetic lethalities and drug combinations that are able to eliminate drug resistant cells.

This work reports the development of four DOX-resistant models of bone sarcoma (three osteosarcomas and one chondrosarcoma), including a pioneer model generated from a patient-derived chondrosarcoma cell line. We performed a comprehensive functional in vitro and in vivo characterization of these models. Altogether, we proved the utility of these drug-resistant cells as models to study the mechanisms underlying the resistance to DOX in bone sarcomas.

## 2. Results

### 2.1. Establishment of DOX-Resistant Bone Sarcoma Cell Lines and Characterization of Their Re-sponse to Other Anti-Tumor Drugs

**Figure 1:**
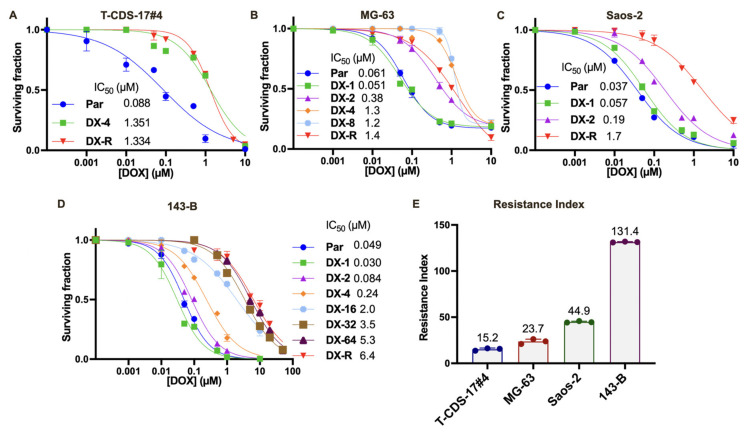
Establishment of DOX-resistant (DX-R) bone sarcoma cell lines. (**A**–**D**) Cell viability (WST-1 assay) measured after different rounds of DOX treatment to verify acquired resistance over time. IC_50_ values for each condition are shown. (**E**) Final Resistance Index (RI) achieved of each DX-R bone sarcoma cell line established.

A panel of four bone sarcoma cell lines (three osteosarcoma lines (143-B, Saos-2 and MG-63) and one primary-derived chondrosarcoma line (T-CDS-17#4)) were exposed to several rounds of stepwise increasing concentrations of DOX in order to induce/select resistant phenotypes (Table 1). To monitor the process of resistance acquisition, we calculated IC_50_ values for each cell line after each round of treatment (Figure 1A–D). After periods of drug exposure between 3 and 8 months, all cell lines developed a stable resistance with resistance index (RI) values ranging from 15 for T-CDS-17#4 cells to 131 for the 143-B cell line (Figure 1E) (Table 1).

**Figure 2:**
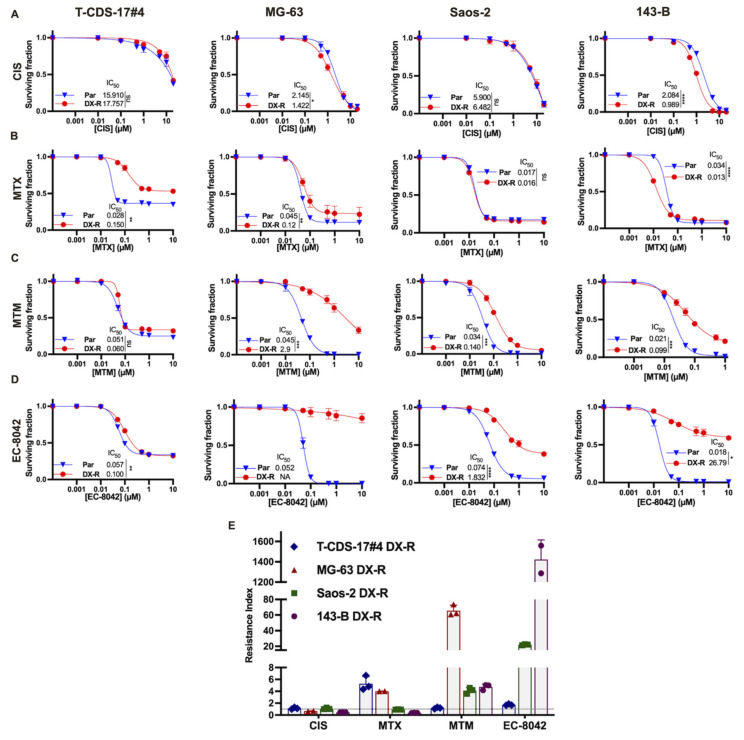
Cross-resistance of DX-R bone sarcoma cell lines to other chemotherapeutic drugs. (**A**–**D**) Cell viability (WST-1 assay) measured after the treatment of parental and DX-R bone sarcoma cell lines with increasing concentrations of cisplatin (CIS) (**A**), methotrexate (MTX) (**B**), mithramycin (MTM) (**C**) and EC-8042 (**D**) for 72 h. IC_50_ values (µM) for each condition are shown. Error bars represent the SD. Asterisks indicate statistically significant differences between IC_50_ concentrations calculated from three independent experiments (ns: *p* > 0.05; *: *p* < 0.05; **: *p* < 0.01; ***: *p* < 0.001; ****: *p* < 0.0001; two-sided Student’s *t* test). (**E**) Resistance index (RI) for the different drugs calculated as the ratio of the IC_50_ values of the resistant cells and the corresponding parental cells. The dotted line represents a resistance index of 1, which indicates equal sensitivity in DX-R and parental cells.

By comparing the cell survival curves of parental and DOX-resistant (DX-R) cells, we analyzed whether DOX-resistance induced a higher tolerance or sensitivity to other compounds with reported anti-tumor activity against sarcoma cells, such as cisplatin (CIS), methotrexate (MTX), mithramycin (MTM) and EC-8042 (Figure 2). First, we found that DOX-resistant cells displayed differential responses to CIS. T-CDS-17#4 cells showed a poor response to this drug, regardless of their DOX-resistance status. Among the other three osteosarcoma cell lines, we found significant differences in the response to CIS in the case of 143-B-DX-R and MG-63-DX-R cells, which were slightly more sensitive (IC_50_ two times lower) than their parental counterparts (Figure 2A,E). No clear trends were seen for MTX either. Thus, 143-B-DX-R cells were more sensitive (IC_50_ three times lower), MG-63-DX-R and T-CDS-17#4-DX-R were more resistant (IC_50_ three and five times higher, respectively) and no differences were found in the response of parental and resistant strains of Saos-2 cells (Figure 2B,E). Finally, MTM and its derivative EC-8042 induced anti-proliferative effects in all parental cells (IC_50_′s ranging from 21 (143-B) to 51 nM (T-CDS-17#4) for MTM and from 18 (143-B) to 74 nM (Saos-2) for EC-8042). With the only exception of TCDS-17#4 for MTM, all resistant lines showed increased resistance to both drugs (IC_50_ for MTM between 4 and 64 times higher and IC_50_ for EC-8042 between 2 and more than 1000 times higher) (Figure 2C–E).

### 2.2. Differential Phenotypes of DOX-Resistant Bone Sarcoma Cells

**Figure 3:**
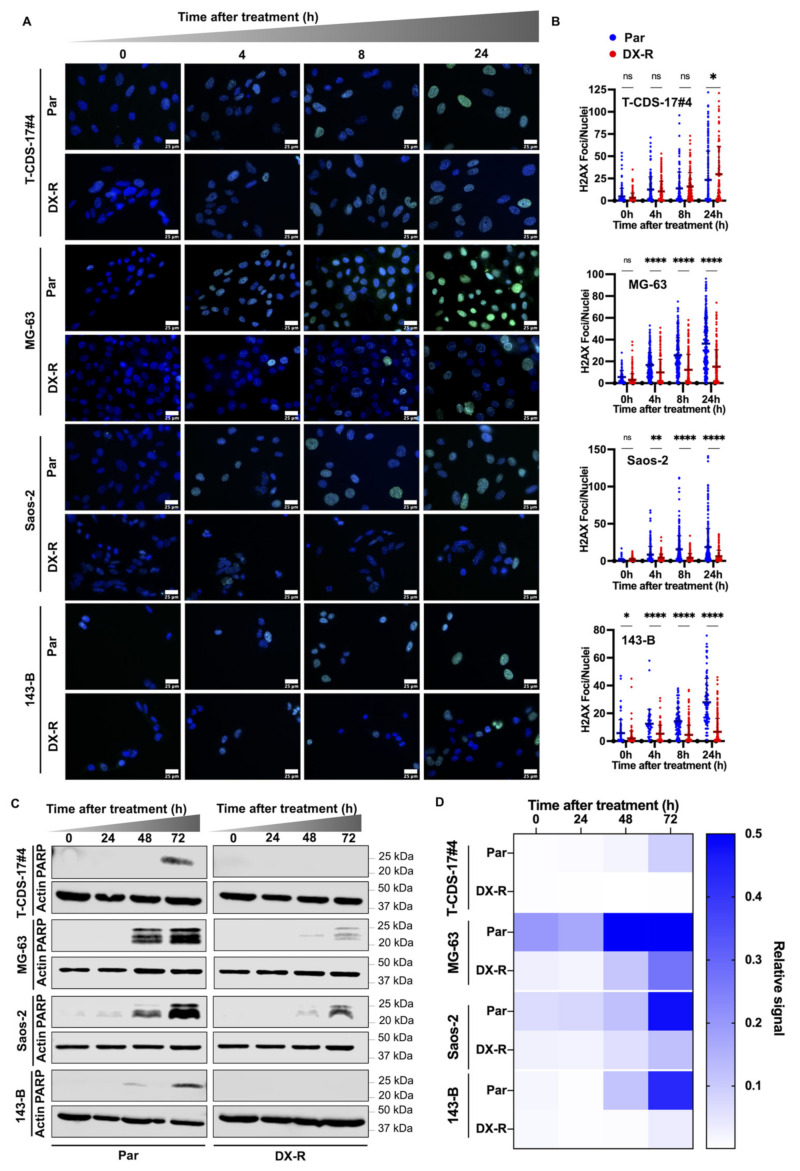
Induction of DNA damage and apoptosis in DX-R bone sarcoma models. (**A**,**B**) Analysis of the formation of γ-H2AX foci in parental and DX-R bone sarcoma cell lines after treatment with 1 µM DOX for 0 h (control), 4 h, 8 h or 24 h. (**A**) Representative images of immunostaining experiments (γ-H2AX immunodetection: green fluorescence; DAPI staining: blue fluorescence) for each condition. Scale bars = 25 µm. (**B**) Quantification of the γ-H2AX foci. Means (horizontal bars) and SD of the number of foci of at least 100 cells for each condition are shown. Asterisks indicate statistically significant differences between groups in one-way ANOVA test (ns: *p* > 0.05; *: *p* < 0.05; **: *p* < 0.01; ****: *p* < 0.0001). (**C**) Western blotting analyses of cleaved PARP1 (PARP) levels in parental and DX-R bone sarcoma cell lines treated with their respective IC_50_ concentrations of DOX (90 nM for T-CDS-17#4, 61 nM for MG-63, 37 nM for Saos-2 and 50 nM for 143-B) for the indicated times. β -Actin levels were used as loading controls. (**D**) Quantification of the Western blotting analyses. Protein levels were normalized to β-actin.

To gain insight into the resistant phenotype, we compared the effect of DOX in the induction of DNA damage and the triggering of apoptosis in parental and resistant lines. As expected, we found that DOX was a potent inducer of DNA damage, as indicated by the time-dependent accumulation of γ-H2AX foci in parental lines. Remarkably, the level of DNA damage detected in the resistant strains of the osteosarcoma cell lines 143-B and Saos-2 was significantly lower than those of the parental cells, while no differences between parental and resistant cells were found in the chondrosarcoma line T-CDS-17#4 (Figure 3A,B).

Next, we analyzed the protein levels of a cleaved form of PARP1, a substrate that is processed by caspase 3 upon the onset of apoptosis, to estimate the induction of apoptosis after DOX treatment. Time course experiments showed that PARP1 was efficiently processed in all parental cell lines treated with their respective IC_50_ concentrations of DOX. As expected, the levels of cleaved PARP1 were barely increased, or not detected, in all resistant lines after 72 h of treatment with DOX (Figure 3C,D).

**Figure 4:**
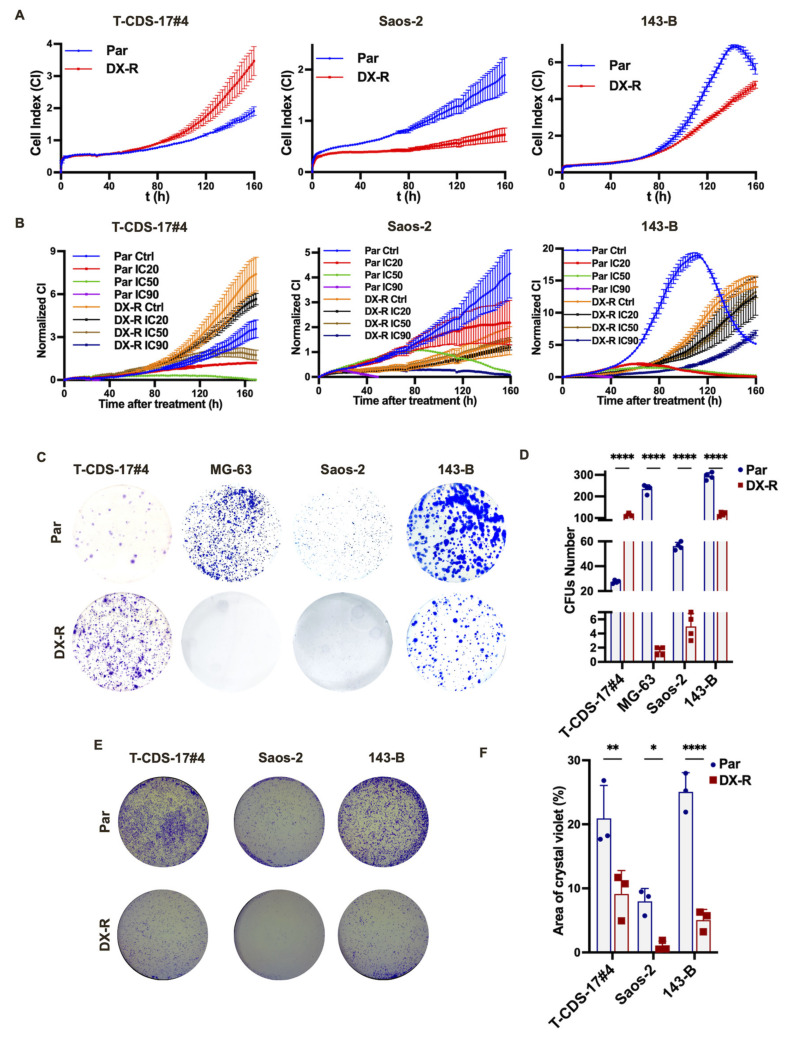
Proliferative and migration capability of DX-R bone sarcoma cell lines. (**A**,**B**) Real-time proliferation analysis (iCELLigence system) of the indicated parental and DX-R cells in the absence (**A**) or presence of IC_20_ (8 nM for T-CDS-17#4, 6 nM for Saos-2 and 10 nM for 143-B), IC_50_ (90 nM for T-CDS-17#4, 37 nM for Saos-2 and 50 nM for 143-B) or IC_90_ (4600 nM for T-CDS-17#4, 633 nM for Saos-2 and 350 nM for 143-B) concentration of DOX (**B**). (**C**,**D**) Colony formation unit (CFU) assay of parental and DX-R bone sarcoma cell lines. Representative images (**C**) and quantification of the number of colonies obtained in each case (**D**) are shown. (**E**,**F**) Transwell migration assay of parental and DX-R bone sarcoma cell lines. Representative images (**E**) and quantification of the surface occupied by migrated cells (**F**) are shown. Data represent the mean and SD of three independent experiments. Asterisks indicate statistically significant differences between groups in one-way ANOVA test (ns: *p* > 0.05; *: *p* < 0.05; **: *p* < 0.01; ****: *p* < 0.0001).

To further characterize the phenotype associated with DOX resistance in bone sarcoma cells we used an iCELLigence system to compare the proliferation capability of parental and DOX-resistant cells. In these analyses we found that all assayed osteosarcoma DOX-resistant cell lines (143-B-DX-R and Saos-2-DX-R) proliferated slower than their parental counterparts. However, the chondrosarcoma resistant line T-CDS-17#4-DX-R showed the opposite trend and grew faster than its parental line (Figure 4A). In addition, iCELLigence analyses also showed that DX-R cell lines were able to continue proliferating in the presence of concentrations of doxorubicin that efficiently eliminated parental cells (Figure 4B). Colony formation unit (CFU) assays showed that, in agreement with their proliferation capability, 143-B-DX-R, Saos-2-DX-R and MG-63-DX-R cells were less clonogenic than their respective parental cells, while T-CDS 17#4-DX-R cells showed a higher clonogenic ability than their parental cell line (Figure 4C,D). Finally, transwell migration assays showed that all assayed DOX-resistant cells exhibited a reduced migration capability compared to their respective parental cells (Figure 4E,F).

Altogether, these results showed that bone sarcoma DOX-resistant cells show some common phenotypic traits, such as their ability to tolerate the DNA damaging and pro-apoptotic effects of doxorubicin or their relatively lower migratory potential. However, other phenotypic features, such as proliferative and clonogenic potential, seem to be case- and/or tumor type-dependent.

### 2.3. Stemness-Related Phenotypes in DOX-Resistant Bone-Sarcoma Cell Line

**Figure 5:**
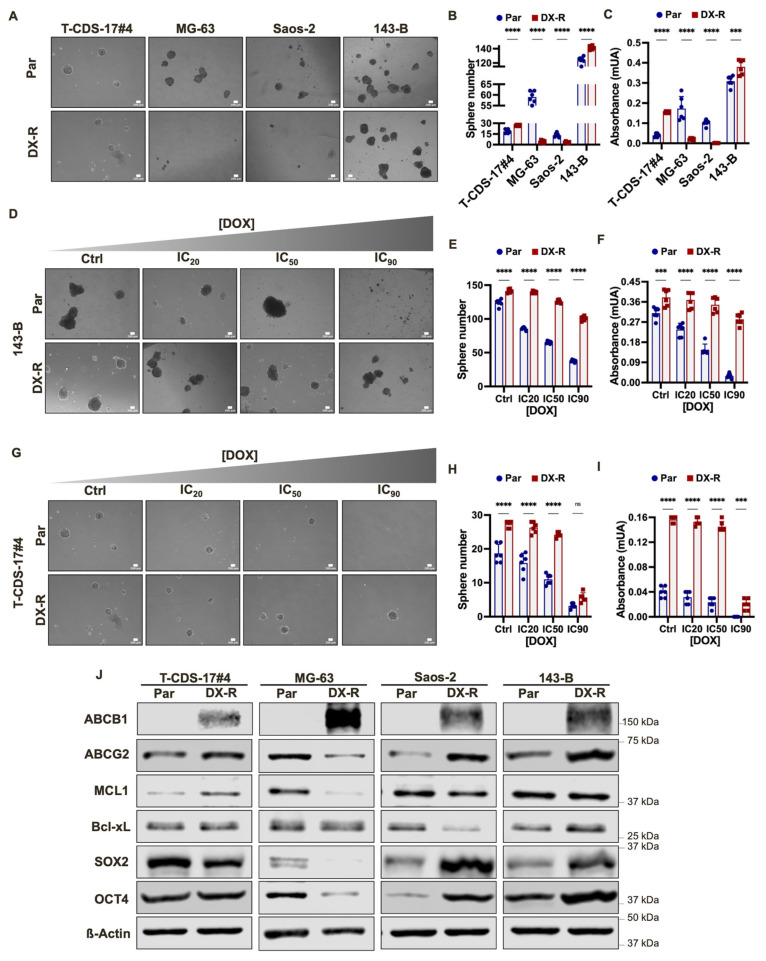
Stemness-related phenotypes in DX-R bone-sarcoma cell lines. (**A**–**C**) Tumorsphere formation potential of parental and DX-R bone sarcoma cell lines. Representative images of each condition (**A**) and quantification of the number of tumorspheres by direct counting (**B**) and cell viability (WST-1 assays) (**C**) are presented. (**D**–**I**) Effect of DOX treatment on the viability of tumorsphere cultures of parental and DX-R 143-B (**D**–**F**) and T-CDS-17#4 cells (**G**–**I**). Tumorsphere 10-day cultures were treated with IC_20_ (10 nM for 143-B and 8 nM for T-CDS-17#4), IC_50_ (50 nM for 143-B and 90 nM for T-CDS-17#4) or IC_90_ (350 nM for 143-B and 4600 nM for T-CDS-17#4) concentrations of DOX for 96 h. After this period, representative images were taken (**D**,**G**) and the effect of the drug on tumorsphere cultures was examined through the counting of the remaining tumorspheres (**E**,**H**) and the analysis of cell viability (WST-1 assays) (**F**,**I**). Scale bars = 200 µm. Data represent the mean and SD of three independent experiments. Asterisks indicate statistically significant differences between groups in one-way ANOVA test (ns: *p* > 0.05; ***: *p* < 0.001; ****: *p* < 0.0001). (**J**) Western blotting analyses of the indicated stem cell and drug-resistance related factors in parental and DX-R bone sarcoma cell lines. β -Actin levels were used as loading controls.

The development of drug resistance by tumor cells has often been linked to the acquisition of a stemness phenotype [23]. Therefore, we analyzed the ability of parental and DOX-resistant cells to form clonal tumorspheres in anchorage independent conditions. The ability to grow in these conditions was associated with the self-renewal and tumorigenic potential of CSCs in many tumor types, including sarcomas [20,22,35]. These assays showed a wide range of variability in the tumorsphere-forming potential of the parental lines, the most and least sphere-forming lines being 143-B and Saos-2 cells, respectively. Among DOX-resistant lines, 143-B-DX-R and T-CDS-17#4-DX-R cells formed tumorspheres more efficiently than their parental lines, while Saos-2-DX-R and MG-63-DX-R cells formed less tumorspheres than their respective parental strains (Figure 5A–C). In any case, as seen in adherent conditions, tumorsphere cultures of different DOX-resistant lines, such as 143-B-DX-R and T-CDS 17 #4-DX-R, were significantly less sensitive to DOX than parental cells (Figure 5D–I).

To further investigate the CSC phenotype in doxorubicin-resistant cells, we checked the protein levels of relevant factors associated with the drug resistant and/or stemness phenotypes. These analyses showed that the resistant phenotype was associated with the upregulation of the efflux pumps ABCB1 and ABCG2. Thus, while ABCB1 was not detected in any parental line, it was strongly upregulated in all resistant lines. Similarly, with the exception of MG-63-DX-R, we found increased levels of ABCG2 in most resistant cell lines (Figure 5J). On the other hand, despite the clear relationship between the resistant phenotype and poor apoptotic responses, we did not find a clear trend regarding expression of key anti-apoptotic factors of the BCL-2 family. We only found slight increases in the levels of BCL-xL in 143-B-DX-R cells and MCL-1 in T-CDS-17#4-DX-R cells. In contrast, both anti-apoptotic factors were downregulated in Saos-2-DX-R and MG-63-DX-R cells (Figure 5J). Finally, we found that the pluripotency factors SOX-2 and OCT-4 were upregulated in the osteosarcoma resistant lines 143-B-DX-R and Saos-2-DX-R, compared to their parental cells. In contrast, these pluripotency factors were heavily downregulated in the osteosarcoma line MG-63-DX-R, and the chondrosarcoma line T-CDS-17#4-DX-R showed lower SOX-2 levels than its parental cell line (Figure 5D).

### 2.4. DOX-Resistant Cells Maintain Resistance In Vivo

**Figure 6:**
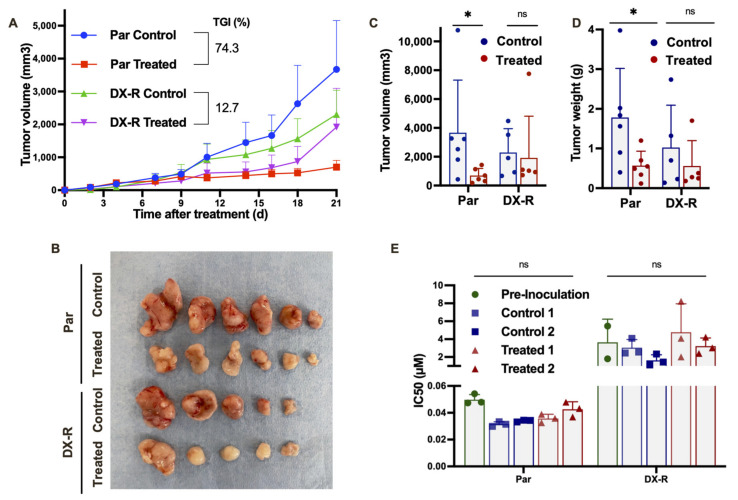
143-B-DX-R cells maintain resistance in vivo. Mice carrying tumor xenografts generated by parental 143-B or 143-B-DX-R cells were randomly assigned to two different groups (*n* = 6 per group) and treated i.v. either with saline buffer (control) or DOX at a dose of 4 mg/Kg twice a week (7 doses). (**A**) Curves representing the mean relative tumor volume of the different groups during the treatments. Drug efficacy is expressed as the percentage of TGI at the end of the experiment. Mean ± SEM is presented. (**B**) Image of the tumors extracted from the mice at the end of the experiment. (**C**,**D**) Distribution of tumor volumes (**C**) and weights (**D**) at the end of the experiment. (**E**) Comparison of the cytotoxic effect of DOX (IC_50_ values) in parental 143-B and 143-B-DX-R cell lines before (pre-inoculation) and after (post-inoculation) in vivo tumor growth in immunodeficient mice. Error bars represent the SD and asterisks indicate statistically significant differences between groups in one-way ANOVA test (ns: *p* > 0.05; *: *p* ≤ 0.05).

To assay whether the resistant phenotype is maintained in an in vivo setting, we generated tumor xenografts by inoculating parental and DOX-resistant 143-B cells subcutaneously in immunodeficient mice and treated them either with the drug vehicle (control) or 4 mg/Kg doxorubicin twice a week. In control conditions, parental cells were more tumorigenic and produced larger tumors than DOX-resistant cells (Figure 6A,B). In agreement with in vitro experiments, DOX treatment was able to efficiently reduce tumor growth in xenografts generated by parental cells, but was much less effective in those generated by resistant cells. At the experimental end-point, parental cells treated with DOX showed a tumor growth inhibition percentage (%TGI) of 74.3%, while resistant cells treated with this drug reduced their growth by only 12.7% (Figure 6A,B). In addition, we found statistically significant differences between the volumes and weights of tumors of control and treated series in xenografts generated by parental, but not by resistant, cells (Figure 6C,D).

To examine whether the level of resistance was affected by in vivo growth, we derived cell lines from tumors of all conditions and treated them in vitro with increasing concentrations of doxorubicin. In these cell viability assays, we found that both parental and resistant lines maintained IC_50_ values similar to those obtained before xenograft growth, thus, confirming the stability of the resistance phenotype (Figure 6E).

Taken together, these data demonstrate feasibility of using these in vitro-generated DOX-resistant lines as models of resistance in in vivo and ex vivo settings.

## 3. Discussion

Bone sarcomas are highly aggressive tumors that typically affect children and young adults. Chemotherapeutic drugs, such as DOX, remain the most relevant treatment to control the disease, even though drug resistance can be a strong limitation [9,36]. Therefore, research efforts should be intensified to develop improved preclinical models useful for deciphering new molecular mechanisms involved in the development of the resistant phenotype. To contribute to this objective, we have developed and functionally characterized a panel of four bone sarcoma cell lines resistant to DOX. These models expand the still scarce panel of DOX-resistant osteosarcoma and chondrosarcoma cell lines available [14,23,37,38,39,40,41]. To the best of our knowledge, this is the first study describing the generation of DOX-resistant models developed from a chondrosarcoma patient-derived cell line. Unlike a previous study that had a low success rate (17%) in establishing DOX-resistant osteosarcoma cell lines [39], our protocol was able to achieve resistance in all four cell lines assayed.

Our phenotypic characterization of the new resistant cell lines revealed the existence of both common and cell line-specific features. Among common phenotypic traits shared by both osteosarcoma and chondrosarcoma models, we found that resistant models showed an increased ability to tolerate the DNA damaging and pro-apoptotic effects of doxorubicin. In addition, all resistant models displayed a lower migratory potential. Other phenotypic traits seem to be tumor type-dependent. Thus, we found that all osteosarcoma resistant lines were less proliferative, both in vitro and in vivo, and less clonogenic than their corresponding parental lines. However, the opposite trend was observed in the resistant chondrosarcoma T-CDS-17#4-DX-R cell line. The lower proliferative index of resistant osteosarcoma models could be associated with the acquisition of a quiescent/dormant state, which may allow tumor cells to better tolerate chemotherapeutic drugs [21,42]. A similar decrease in the proliferative, clonogenic and migratory potentials has also been reported for other osteosarcoma DOX-resistant models [39,43,44]. Therefore, it is plausible that the adoption of a lower proliferative/migratory phenotype could be a prevalent mechanism used by osteosarcoma cells to acquire resistance to DOX treatment. In addition, CSC subpopulations have been reported as being a key factor in chemoresistance [23]. Here we found that there are both DOX-resistant models, that showed an increased ability to form tumorspheres and a higher expression of pluripotency factors, and other models in which we found the opposite effect. These data suggest that DOX-resistance in bone sarcomas is not always associated with the adoption of CSC-related phenotypes and, in certain cases, CSC-independent mechanisms may be driving the development of resistance.

The most widely described mechanism of resistance to DOX and other chemotherapeutic drugs is the overexpression of ABCB1 and other members of the ABC family of transporters, which leads to increased efflux and decreased intracellular accumulation [11,15,16,43]. According to these reports, we found that ABCB1 was strongly upregulated in all DOX-resistant models. Other work has correlated the expression of other ABC transporters, such as ABCG1, with the self-renewal potential of osteosarcoma DOX-resistant cells [41]. In a similar way, we found that ABCG2 was upregulated in the DOX-resistant osteosarcoma lines that showed an increased expression of SOX-2 and OCT-4 (143-B-DX-R and Saos-2-DX-R), but downregulated in the DOX-resistant line which displays a repressed expression of the pluripotency factors (MG-63-DX-R). In summary, these results suggest that, while the overexpression of ABCB1 is a common mechanism of drug resistance, the expression of ABCG2 could be exclusively associated with CSC-mediated resistance.

The upregulation of anti-apoptotic factors of the BCL-2 family has also been associated with DOX resistance in bone sarcomas [17,18]. We did not find a clear trend regarding the expression of BCL-xL and MCL-1 in the resistant models, wherein MCL-1 in T-CDS-17#4-DX-R cells were the most prevalent upregulated anti-apoptotic factor. Given that T-CDS-17#4 is the model that showed lower levels of resistance and lower expression of ABC pumps, it could be speculated that the development of anti-apoptotic mechanisms is associated with the first steps in the development of a resistant phenotype, while ABC transporters could play a more relevant role in the later phases of this process.

All osteosarcoma DOX-resistant lines were also highly resistant to treatment with MTM and its related analog EC-8042, thus suggesting the existence of common mechanisms of resistance between these compounds and DOX. However, osteosarcoma DOX-resistant models did not show an increased resistance to CIS or MTX, and even the most DOX-resistant model (143-B-DX-R) became slightly more sensitive to both CIS and MTX. In agreement with this finding, no cross-resistance to CIS or MTX was observed in other previously reported DOX-resistant osteosarcoma cell lines [39]. Although resistance to CIS and MTX has been frequently associated with the efflux activity of ABC pumps [19], these results suggest that this is not always the case and that in osteosarcomas there could be differential mechanisms of resistance to DOX and CIS or MTX. Thus, it is plausible that a chronic exposure of osteosarcoma cells to DOX does not always affect other mechanisms of resistance to MTX, such as modulation of the expression of MTX target dihydrofolate reductase (DHFR), or to CIS, such as the deregulation of BER-/NER-mediated DNA repair or the inactivation trough detoxification enzymes, like GSTP1-1 [19,45,46]. Relevant to the clinic, the increased sensitivity to methotrexate and cisplatin observed in doxorubicin-resistant cells could be exploited for the election of alternative treatments for osteosarcoma patients upon the development of doxorubicin resistance [36].

Importantly, the treatment of xenograft models confirmed that the resistance phenotype of the 143-B-DX-R model is also maintained in the in vivo setting, thus allowing the use of this cell line as a drug testing model suitable for in vivo assessment of the effectiveness of therapies aimed to circumvent doxorubicin resistance.

Altogether, the characterization of four DOX-resistant bone sarcoma models evidenced that there may be different phenotypes and mechanisms associated with the development of resistance to DOX. Anyway, this is an initial phenotypic characterization of these bone sarcoma resistant models. It is foreseen that further molecular characterization of these models, integrating proteomic, transcriptomic, epigenetic and/or metabolomics analyses together with other approaches, such as CRISPR screening assays, will contribute to the unravelling of the molecular mechanisms behind common and specific phenotypic traits in resistant cells. These analyses will also result in the identification of synthetic lethalities and drug combinations able to revert doxorubicin resistance in bone sarcoma and/or the identification of biomarkers useful to select those patients which will benefit most from DOX treatment. As future goals, it would also be of interest to compare early versus late stages of resistance in these step-wise generated models. These studies will help to understand the time course of events involved in the process of acquisition of drug resistance by bone sarcoma cells. Besides, this study only included one chrondrosarcoma resistant line. To better discern the resistance mechanisms associated with this type of bone sarcoma, it is necessary to develop and characterize more drug-resistant chondrosarcoma models. Finally, the development of drug resistance in patient-derived models, as reported in this work, may allow the implementation of personalized strategies capable of anticipating efficient second-line treatments in cases of relapse.

## 4. Materials and Methods

### 4.1. Cell Culture

Human osteosarcoma cell lines 143-B (CRL-8303), Saos-2 (HTB-85), and MG-63 (CRL-1427) were obtained from ATCC (Manassas, VA, USA). Patient-derived chondrosarcoma model T-CDS-17#4 was stablished as previously described [47]. All cell lines were cultured in Dulbecco’s modified Eagle medium (DMEM, Corning, AZ, USA) supplemented with 10% (*v*/*v*) fetal bovine serum (FBS, Gibco, Carlsbad, CA, USA), 1% (*v*/*v*) Penicillin-Streptomycin (10,000 U/mL; Gibco, Carlsbad, CA, USA) and 1% (*v*/*v*) GlutaMAX (Gibco, Carlsbad, CA, USA) at 37 °C in a humidified atmosphere (5% CO_2_ and 95% air). All cultures were tested monthly to discard mycoplasma contamination using the Biotools Mycoplasma Gel Detection kit (B&M LABS, Madrid, Spain).

All experimental protocols have been performed in accordance with institutional review board guidelines and with the Declaration of Helsinki and were approved by the Institutional Ethics Committee of the Principado de Asturias (reference 255/19).

### 4.2. Drugs

DOX, MTX and CIS were purchased from Selleckchem (Houston, TX, USA). MTM and its analog EC-8042 were synthesized by EntreChem SL (Oviedo, Spain) [48,49]. Stocks were prepared as 10 mM solutions in sterile DMSO (DOX, MTX, MTM and EC-8042) or DMF (CIS), stored at −80 °C and diluted in culture medium to the final concentration just before use.

### 4.3. Generation of DOX-Resistant Cell Lines

Sarcoma DOX-resistant (DX-R) cell lines were generated by continuous exposure to stepwise increasing concentrations of DOX. Briefly, the parental cell lines were continuously treated with their respective IC_20_ of DOX (calculated after 72 h-treatments). The drug was renewed every 3–4 days in fresh culture medium. After one month, the drug concentration was doubled and cells were cultured for another month. The whole process of step-by-step concentration increases and incubation times was repeated several times until we obtained a resistance level for each cell line that could not be increased with further increases in drug concentration. The conditions used to generate resistant models for each cell line are summarized in Table 1. In most steps, the concentration of half-maximal inhibition of viability (IC_50_) and the resistance index (RI), calculated as the ratio between the IC_50_′s of resistant and parental lines, were determined.

### 4.4. Cell Viability Assays

Cell viability of cell lines after the treatment with increasing concentrations of different drugs was assayed using the cell proliferation reagent WST-1 (Roche, Mannheim, Germany) after 72 h-treatments as previously described [34,50]. IC_50_ for each treatment was determined by non-linear regression using GraphPad Prism 9.0.1 software (Graphpad Software Inc., San Diego, CA, USA).

### 4.5. Real-Time Proliferation Analysis

The differences in the proliferation potential between the DOX-resistant cell lines and their respective parental cells were evaluated using the iCELLigence real-time cell analyzer (ACEA Biosciences, San Diego, CA, USA) [51]. Cells (between 2 × 10^3^ and 1.5 × 10^4^ cells depending on the assayed cell line) were seeded in specially designed 8-well plates (E-plate L8, ACEA Biosciences) that contained interdigitated gold microelectrodes sensors able to detect changes in cell impedance. In experiments designed to analyze the effect of DOX on cell proliferation, this drug was added 6 h after cell seeding. Cell impedance was monitored every two hours for 160 h and data was analyzed using the RTCA analysis software version 1.0 (ACEA Biosciences). The proliferation status of cells was expressed in terms of cell index (CI) normalized or not to the values measured 6 h after seeding.

### 4.6. Colony Formation Unit (CFU) Assay

The clonogenic capability of parental and DX-R cell lines was analyzed in colony formation unit (CFU) assays as described previously [32]. Briefly, cells were seeded at low density (1 × 10^3^ cells) in 100 mm culture dishes and left to form colonies for 10 days. Following this period, cells were fixed in cold methanol and stained with 0.5% crystal violet. Colonies containing approximately 50 or more cells were counted using the ImageJ 2.1.0 software (NIH, Bethesda, MD, USA).

### 4.7. Transwell Migration Assay

Cells were resuspended in a serum-free culture media and seeded in transwell inserts with a 8 µm pore size membrane (upper chamber; Costar, NY, USA) placed in 24-well plates (lower chamber). Plating cell densities were 2.5 × 10^4^ cells in 100 µL per well for 143-B and 5 × 10^4^ cells/well for the rest of cell lines. Then, 600 µL of medium was added to the lower chamber. After 24 h, cells were fixed in 70% ethanol for 10 min and stained with 0.5% crystal violet for 5–10 min. Then, cells on the upper side of inserts were removed with cotton-tipped swabs and the inserts were washed with distilled water [52]. Cells at the bottom of the insert membranes were examined under a stereo microscope and the area of membrane stained with crystal violet (proportional to the number of migrated cells) was quantified using the ImageJ 2.1.0 software (National Institutes of Health, Bethesda, MD, USA).

### 4.8. Tumorsphere Culture

Cells were resuspended in a serum-free sphere-forming medium containing Ham’s-F12 (Corning, NY, USA) supplemented with 1% (*v*/*v*) Penicillin-Streptomycin (10,000 U/mL; Gibco, Carlsbad, CA, USA), 1% (*v*/*v*) GlutaMAX (Gibco, Carlsbad, CA, USA), 2% (*v*/*v*) B-27 Supplement minus vitamin A (50X; Gibco, Carlsbad, CA, USA), 20 ng/mL human EGF (PeproTech, London, UK) and 10 ng/mL human bFGF (PeproTech, London, UK) were seeded at low density (1.5 × 10^3^ cells per well) in 6-well plates treated with a sterile solution of poly 2-hydroxyethyl methacrylate (10 g/L in 95% ethanol; Sigma-Aldrich, St Louis, MO, USA) to prevent cell attachment. Fresh aliquots of EGF and bFGF were added twice a week. Well-rounded spheres formed after 10 days of culture were counted and cell viability was determined using the cell proliferation reagent WST-1 (Roche, Mannheim, Germany). To analyze the effects of DOX on tumorsphere cultures, 10 day-spheres were incubated in a sphere medium containing different concentrations of the drug for 4 days prior to quantifying the number of spheres and relative viability (WST1 assay) in each case [20,34].

### 4.9. Western Blotting

Cell extracts were lysed with Pierce RIPA buffer (Thermo Scientific, Rockford, IL, USA) supplemented with protease and phosphotase inhibitors. Lysates were centrifuged at 12,000 rpm at 4 °C and supernatants were collected. Protein concentration was quantified using the Bradford dye-binding method (Bio-Rad protein assay kit; Bio-Rad Laboratories, Inc., Hercules, CA, USA). A total of 30 µg of protein sample was loaded on 6–10% sodium dodecyl sulfate (SDS)-polyacrylamide gels. After electrophoresis, gels were electro-transferred using the Trans-Blot turbo transfer system and the Trans-Blot turbo RTA mini 0.2 µm nitrocellulose transfer kit (Bio-Rad Laboratories, Inc., Hercules, CA, USA). The membranes were blocked with SuperBlock^TM^ blocking buffer (Thermo Scientific, Rockford, IL, USA) and incubated overnight at 4 °C with primary antibodies diluted in 3% bovine serum albumin (BSA; VWR, Radnor, PA, USA). Primary antibodies used in these analyses were as follows: anti-ABCB1 [[13342], 1:1000 dilution] from Cell Signaling (Danvers, MA, USA); anti-ABCG2 ([ab108312], 1:1000 dilution) and anti-Cleaved-PARP1 ([ab32064], 1:1000 dilution) from Abcam (Cambridge, UK); anti-OCT4 ([11236-1-AP], 1:1000 dilution), anti-SOX2 ([66411-1-Ig], 1:1000 dilution), anti-MCL1 ([16225-1-AP], 1:1000 dilution) and anti-Bcl-xL ([26967-1-AP], 1:1000 dilution) from Proteintech (Wuhan, China); and anti-β-Actin ([A5441], 1:5000 dilution) from Sigma-Aldrich. IRDye infrared fluorescent secondary antibodies IRDye 800CW and IRDye-680RD from LI-COR Biosciences (1:10,000, Lincoln, NE, USA) were used for signal detection using an Odyssey Fc imaging system and the Image Studio software (LI-COR Biosciences, Lincoln, NE, USA). β -actin levels were used as loading control.

### 4.10. Immunofluorescence Staining

Cells were seeded at high density in 6-well plates containing glass coverslips. After 24 h, cells were treated with 1 µM DOX for 0 h (Control), 4 h, 8 h or 24 h. Then, cells were washed twice with phosphate-buffered saline (PBS; Corning, NY, USA) and fixed in 4% paraformaldehyde (PFA; Sigma-Aldrich, Saint-Louis, MO, USA) in PBS for 20 min at room temperature. Cells were then washed twice with PBS plus 0.1% Tween 20 (Sigma-Aldrich), permeabilized in PBS plus 0.1% Triton X-100 (Sigma-Aldrich) for 20 min and washed again with PBS. Slides were blocked in 10% BSA (VWR, Radnor, PA) for 30 min and incubated overnight at 4 °C with the primary antibody anti-γ-H2AX ([05–636], 1:500 dilution) from Merck KGaA (Darmstadt, Germany). Then, the slides were washed 3 times with PBS plus 0.1% Tween 20 and incubated with the secondary antibody Alexa Fluor 488 goat anti-mouse IgG ([A-11001], 1:1000 dilution) from Invitrogen (Waltham, MA, USA) for 1 h. Later, cells were washed another 4 times with PBS and, finally, coverslips were mounted in ProLong^®^ Gold Antifade Mounting medium with DAPI (Life Technologies, Carlsbad, CA, USA) and analyzed by fluorescence microscopy. The number of γ-H2AX foci per nuclei was counted in at least 100 cells of each condition using the ImageJ 2.1.0 software and the Cell Profiler 4.2.1 (Broad Institute, Cambridge, MA, USA) [53].

### 4.11. In Vivo Tumor Growth

All animal research protocols were carried out in accordance with the institutional guidelines of the University of Oviedo and were approved by the Animal Research Ethical Committee of the University of Oviedo prior to the study (PROAE 34/2019). Female 6 weeks old athymic nude mice (Envigo, Barcelona, Spain) were inoculated subcutaneously (s.c.) with 1 × 10^5^ 143-B parental or DOX-resistant cells resuspended 1:1 in 50% (*v*/*v*) Matrigel^®^ basement membrane matrix high concentration (Corning, NY, USA). Once tumors reached approximately 100 mm^3^, the mice were randomly assigned (*n* = 6 per group) to receive the following intravenous treatments twice a week (7 doses): vehicle (saline; B. Braun, Melsungen, Germany) or 4 mg/Kg DOX. Tumor volume was determined using a caliper as previously described [30]. Relative tumor volume (RTV) was defined as the difference between the tumor volume at day of measurement and (V_t_) and the tumor volume at the beginning of the treatment (V_0_). Drug efficacy was expressed as the percentage tumor growth inhibition (%TGI), calculated as follows: TGI (%) = 100 − (T/C × 100), where T is the mean RTV of the treated tumor and C is the mean RTV in the control group at day of measurement. Three weeks after starting the treatment, mice were sacrificed by cervical dislocation and tumors were extracted. Cell lines were stablished from representative tumors from mice of different experimental groups as described before [47]. WST-1 assays were performed to check the resistance status of these ex-vivo cell lines.

### 4.12. Statistical Analysis

Statistical analysis was performed using GraphPad Prism 9.0.1 software. Unless otherwise indicated, all data are presented as the mean (±standard deviation or SEM as indicated) of at least three independent experiments. Two-sided Student’s *t* test was performed to determine the statistical significance between groups. Multiple comparisons of the data were performed using one-way ANOVA and Tukey’s test, *p* ≤ 0.05 values were considered statistically significant.

## Figures and Tables

**Table 1:** Generation of DOX-resistant bone sarcoma cell lines.

Cell Line (Tumor Type)	Initial IC_50_ (µM)	Drug-Resistance Induction Protocol								Final IC_50_ (µM)	RI
		Step 1	Step 2	Step 3	Step 4	Step 5	Step 6	Step 7	Step 8		
T-CDS-17#4 (chondros.)	0.088	0.008 µM (IC_20_) 1 month	0.016 µM (2 × IC_20_) 1 month	0.032 µM (4 × IC_20_) 1 month	0.064 µM (8 × IC_20_) 1 month	-	-	-	-	1.3	15.2
MG-63 (osteos.)	0.061	0.019 µM (IC_20_) 1 month	0.038 µM (2 × IC_20_) 1 month	0.076 µM (4 × IC_20_) 1 month	0.152 µM (8 × IC_20_) 1 month	0.304 µM (16 × IC_20_) 1 month	-	-	-	1.4	23.7
Saos-2 (osteos.)	0.037	0.006 µM (IC_20_) 1 month	0.012 µM (2 × IC_20_) 1 month	0.024 µM (4 × IC_20_) 1 month	-	-	-	-	-	1.7	44.9
143-B (osteos.)	0.049	0.010 µM (IC_20_) 1 month	0.020 µM (2 × IC_20_) 1 month	0.040 µM (4 × IC_20_) 1 month	0.080 µM (8 × IC_20_) 1 month	0.160 µM (16 × IC_20_) 1 month	0.320 µM (32 × IC_20_) 1 month	0.640 µM (64 × IC_20_) 1 month	1.280 µM (128 × IC_20_) 1 month	6.4	131.4

Abbreviations: chondros, chondrosarcoma; osteos, osteosarcoma.

## Data Availability

Not applicable.

## References

[1] Heymann D. (2021). Bone Cancer - Bone Sarcomas and Bone Metastasis - From Bench to Bedside.

[2] The WHO Classification of Tumours Editorial Board (2020). WHO Classification of Tumours: Soft Tissue and Bone Tumours.

[3] Alfranca A., Martinez-Cruzado L., Tornin J., Abarrategi A., Amaral T., De Alava E., Menendez P., Garcia-Castro J., Rodriguez R. (2015). Bone microenvironment signals in osteosarcoma development. Cell Mol. Life Sci..

[4] Gambera S., Abarrategi A., Rodríguez-Milla M.A., Mulero F., Menéndez S.T., Rodriguez R., Navarro S., García-Castro J. (2018). Role of Activator Protein-1 Complex on the Phenotype of Human Osteosarcomas Generated from Mesenchymal Stem Cells. Stem Cells.

[5] Kovac M., Blattmann C., Ribi S., Smida J., Mueller N.S., Engert F., Castro-Giner F., Weischenfeldt J., Kovacova M., Krieg A. (2015). Exome sequencing of osteosarcoma reveals mutation signatures reminiscent of BRCA deficiency. Nat. Commun..

[6] Smida J., Xu H.-E., Zhang Y., Baumhoer D., Ribi S., Kovac M., Von Luettichau I., Bielack S., O’Leary V.B., Leib-Mösch C. (2017). Genome-wide analysis of somatic copy number alterations and chromosomal breakages in osteosarcoma. Int. J. Cancer.

[7] Speetjens F.M., de Jong Y., Gelderblom H., Bovée J.V. (2016). Molecular oncogenesis of chondrosarcoma: Impact for targeted treatment. Curr. Opin. Oncol..

[8] Casali P.G., Bielack S., Abecassis N., Aro H.T., Bauer S., Biagini R., Bonvalot S., Boukovinas I., Bovee J., Brennan B. (2018). Bone sarcomas: ESMO-PaedCan-EURACAN Clinical Practice Guidelines for diagnosis, treatment and follow-up. Ann Oncol.

[9] Grünewald T.G., Alonso M., Avnet S., Banito A., Burdach S., Cidre-Aranaz F., Di Pompo G., Distel M., Dorado-Garcia H., Garcia-Castro J. (2020). Sarcoma treatment in the era of molecular medicine. EMBO Mol. Med..

[10] Hulst M.B., Grocholski T., Neefjes J.J.C., van Wezel G.P., Metsä-Ketelä M. (2021). Anthracyclines: Biosynthesis, engineering and clinical applications. Nat. Prod. Rep..

[11] Hattinger C., Patrizio M., Fantoni L., Casotti C., Riganti C., Serra M. (2021). Drug Resistance in Osteosarcoma: Emerging Biomarkers, Therapeutic Targets and Treatment Strategies. Cancers.

[12] Roberts R.D., Lizardo M.M., Reed D.R., Hingorani P., Glover J., Allen-Rhoades W., Fan T., Khanna C., Sweet-Cordero E.A., Cash T. (2019). Provocative questions in osteosarcoma basic and translational biology: A report from the Children’s Oncology Group. Cancer.

[13] David E., Blanchard F., Heymann M.-F., De Pinieux G., Gouin F., Rédini F., Heymann D. (2011). The Bone Niche of Chondrosarcoma: A Sanctuary for Drug Resistance, Tumour Growth and also a Source of New Therapeutic Targets. Sarcoma.

[14] Belisario D.C., Akman M., Godel M., Campani V., Patrizio M.P., Scotti L., Hattinger C.M., De Rosa G., Donadelli M., Serra M. (2020). ABCA1/ABCB1 Ratio Determines Chemo- and Immune-Sensitivity in Human Osteosarcoma. Cells.

[15] Robey R.W., Pluchino K.M., Hall M.D., Fojo A.T., Bates S.E., Gottesman M.M. (2018). Revisiting the role of ABC transporters in multidrug-resistant cancer. Nat. Rev. Cancer.

[16] Serra M., Hattinger C.M., Pasello M., Casotti C., Fantoni L., Riganti C., Manara M.C. (2021). Impact of ABC Transporters in Osteosarcoma and Ewing’s Sarcoma: Which Are Involved in Chemoresistance and Which Are Not?. Cells.

[17] Baranski Z., de Jong Y., Ilkova T., Peterse E.F., Cleton-Jansen A.M., van de Water B., Hogendoorn P.C., Bovee J.V., Danen E.H. (2015). Pharmacological inhibition of Bcl-xL sensitizes osteosarcoma to doxorubicin. Oncotarget.

[18] De Jong Y., Monderer D., Brandinelli E., Monchanin M., Akker B.E.V.D., van Oosterwijk J., Blay J.Y., Dutour A., Bovée J.V.M.G. (2018). Bcl-xl as the most promising Bcl-2 family member in targeted treatment of chondrosarcoma. Oncogenesis.

[19] Marchandet L., Lallier M., Charrier C., Baud’Huin M., Ory B., Lamoureux F. (2021). Mechanisms of Resistance to Conventional Therapies for Osteosarcoma. Cancers.

[20] Cruzado L.M., Tornin J., Santos L., Rodríguez A., Garcia-Castro J., Morís F., Rodriguez R. (2016). Aldh1 Expression and Activity Increase During Tumor Evolution in Sarcoma Cancer Stem Cell Populations. Sci. Rep..

[21] Vallette F.M., Olivier C., Lézot F., Oliver L., Cochonneau D., Lalier L., Cartron P.-F., Heymann D. (2019). Dormant, quiescent, tolerant and persister cells: Four synonyms for the same target in cancer. Biochem. Pharmacol..

[22] Abarrategi A., Tornin J., Martinez-Cruzado L., Hamilton A., Martinez-Campos E., Rodrigo J.P., Gonzalez M.V., Baldini N., Garcia-Castro J., Rodriguez R. (2016). Osteosarcoma: Cells-of-Origin, Cancer Stem Cells, and Targeted Therapies. Stem Cells Int.

[23] Menéndez S., Gallego B., Murillo D., Rodríguez A., Rodríguez R. (2021). Cancer Stem Cells as a Source of Drug Resistance in Bone Sarcomas. J. Clin. Med..

[24] Menendez S.T., Rey V., Martinez-Cruzado L., Gonzalez M.V., Morales-Molina A., Santos L., Blanco V., Alvarez C., Estupiñan O., Allonca E. (2020). SOX2 Expression and Transcriptional Activity Identifies a Subpopulation of Cancer Stem Cells in Sarcoma with Prognostic Implications. Cancers.

[25] Liverani C., Mercatali L., Spadazzi C., La Manna F., De Vita A., Riva N., Calpona S., Ricci M., Bongiovanni A., Gunelli E. (2014). CSF-1 blockade impairs breast cancer osteoclastogenic potential in co-culture systems. Bone.

[26] De Vita A., Vanni S., Miserocchi G., Fausti V., Pieri F., Spadazzi C., Cocchi C., Liverani C., Calabrese C., Casadei R. (2022). A Rationale for the Activity of Bone Target Therapy and Tyrosine Kinase Inhibitor Combination in Giant Cell Tumor of Bone and Desmoplastic Fibroma: Translational Evidences. Biomedicines.

[27] Navet B., Ando K., Vargas-Franco J.W., Brion R., Amiaud J., Mori K., Yagita H., Mueller C.G., Verrecchia F., Dumars C. (2018). The Intrinsic and Extrinsic Implications of RANKL/RANK Signaling in Osteosarcoma: From Tumor Initiation to Lung Metastases. Cancers.

[28] Yamagishi T., Kawashima H., Ogose A., Ariizumi T., Sasaki T., Hatano H., Hotta T., Endo N. (2016). Receptor-Activator of Nuclear KappaB Ligand Expression as a New Therapeutic Target in Primary Bone Tumors. PLoS ONE.

[29] Gaspar N., Venkatramani R., Hecker-Nolting S., Melcon S.G., Locatelli F., Bautista F., Longhi A., Lervat C., Entz-Werle N., Casanova M. (2021). Lenvatinib with etoposide plus ifosfamide in patients with refractory or relapsed osteosarcoma (ITCC-050): A multicentre, open-label, multicohort, phase 1/2 study. Lancet Oncol..

[30] Estupiñan O., Santos L., Rodríguez A., Fernandez-Nevado L., Costales P., Perez-Escuredo J., Hermosilla M.A., Oro P., Rey V., Tornin J. (2018). The multikinase inhibitor EC-70124 synergistically increased the antitumor activity of doxorubicin in sarcomas. Int. J. Cancer.

[31] Montoya S., Soong D., Nguyen N., Affer M., Munamarty S.P., Taylor J. (2021). Targeted Therapies in Cancer: To Be or Not to Be, Selective. Biomedicines.

[32] Estupiñán Ó., Niza E., Bravo I., Rey V., Tornín J., Gallego B., Clemente-Casares P., Moris F., Ocaña A., Blanco-Lorenzo V. (2021). Mithramycin delivery systems to develop effective therapies in sarcomas. J. Nanobiotechnology.

[33] Estupiñán Ó., Rendueles C., Suárez P., Rey V., Murillo D., Morís F., Gutiérrez G., Blanco-López M., Matos M., Rodríguez R. (2021). Nano-Encapsulation of Mithramycin in Transfersomes and Polymeric Micelles for the Treatment of Sarcomas. J. Clin. Med..

[34] Tornin J., Cruzado L.M., Santos L., Rodríguez A., Núñez L.-E., Oro P., Hermosilla M.A., Allonca E., Fernández-García M.T., Astudillo A. (2016). Inhibition of SP1 by the mithramycin analog EC-8042 efficiently targets tumor initiating cells in sarcoma. Oncotarget.

[35] Salerno M., Avnet S., Bonuccelli G., Eramo A., De Maria R., Gambarotti M., Gamberi G., Baldini N. (2013). Sphere-forming cell subsets with cancer stem cell properties in human musculoskeletal sarcomas. Int. J. Oncol..

[36] Hattinger C.M., Fanelli M., Tavanti E., Vella S., Riganti C., Picci P., Serra M. (2017). Doxorubicin-resistant osteosarcoma: Novel therapeutic approaches in sight?. Future Oncol.

[37] Chen J.-C., Huang C., Lee I.-N., Wu Y.-P., Tang C.-H. (2018). Amphiregulin enhances cell migration and resistance to doxorubicin in chondrosarcoma cells through the MAPK pathway. Mol. Carcinog..

[38] Hsieh M., Huang C., Lin C., Tang C., Lin C., Lee I., Huang H., Chen J. (2020). Basic fibroblast growth factor promotes doxorubicin resistance in chondrosarcoma cells by affecting XRCC5 expression. Mol. Carcinog..

[39] da Costa M.E.M., Marchais A., Gomez-Brouchet A., Job B., Assoun N., Daudigeos-Dubus E., Fromigué O., Santos C., Geoerger B., Gaspar N. (2019). In-Vitro and In-Vivo Establishment and Characterization of Bioluminescent Orthotopic Chemotherapy-Resistant Human Osteosarcoma Models in NSG Mice. Cancers.

[40] Rajkumar T., Yamuna M. (2008). Multiple pathways are involved in drug resistance to doxorubicin in an osteosarcoma cell line. Anti-Cancer Drugs.

[41] Roundhill E.A., Jabri S., Burchill S.A. (2019). ABCG1 and Pgp identify drug resistant, self-renewing osteosarcoma cells. Cancer Lett..

[42] Nunes T., Hamdan D., Leboeuf C., El Bouchtaoui M., Gapihan G., Nguyen T.T., Meles S., Angeli E., Ratajczak P., Lu H. (2018). Targeting Cancer Stem Cells to Overcome Chemoresistance. Int. J. Mol. Sci..

[43] Serra M., Scotlandi K., Manara M.C., Maurici D., Lollini P.L., De Giovanni C., Toffoli G., Baldini N. (1993). Establishment and characterization of multidrug-resistant human osteosarcoma cell lines. Anticancer Res..

[44] Yang J.-Z., Ma S.-R., Rong X.-L., Zhu M.-J., Ji Q.-Y., Meng L.-J., Gao Y.-Y., Yang Y.-D., Wang Y. (2016). Characterization of multidrug-resistant osteosarcoma sublines and the molecular mechanisms of resistance. Mol. Med. Rep..

[45] Kanarek N., Keys H.R., Cantor J.R., Lewis C.A., Chan S.H., Kunchok T., Abu-Remaileh M., Freinkman E., Schweitzer L.D., Sabatini D.M. (2018). Histidine catabolism is a major determinant of methotrexate sensitivity. Nature.

[46] Pasello M., Michelacci F., Scionti I., Hattinger C.M., Zuntini M., Caccuri A.M., Scotlandi K., Picci P., Serra M. (2008). Overcoming Glutathione*S*-Transferase P1–Related Cisplatin Resistance in Osteosarcoma. Cancer Res..

[47] Rey V., Menendez S.T., Estupiñan O., Rodriguez A., Santos L., Tornin J., Martinez-Cruzado L., Castillo D., Ordoñez G.R., Costilla S. (2019). New Chondrosarcoma Cell Lines with Preserved Stem Cell Properties to Study the Genomic Drift During In Vitro/In Vivo Growth. J. Clin. Med..

[48] Novakova R., Núñez L.E., Homerova D., Knirschova R., Feckova L., Rezuchova B., Sevcikova B., Menéndez N., Morís F., Cortés J. (2017). Increased heterologous production of the antitumoral polyketide mithramycin A by engineered Streptomyces lividans TK24 strains. Appl. Microbiol. Biotechnol..

[49] Núñez L.E., Nybo S.E., González-Sabín J., Pérez M., Menéndez N., Braña A.F., Shaaban K.A., He M., Morís F., Salas J.A. (2012). A Novel Mithramycin Analogue with High Antitumor Activity and Less Toxicity Generated by Combinatorial Biosynthesis. J. Med. Chem..

[50] Tornin J., Hermida-Prado F., Padda R.S., Gonzalez M.V., Alvarez-Fernandez C., Rey V., Martinez-Cruzado L., Estupinan O., Menendez S.T., Fernandez-Nevado L. (2018). FUS-CHOP Promotes Invasion in Myxoid Liposarcoma through a SRC/FAK/RHO/ROCK-Dependent Pathway. Neoplasia.

[51] Ke N., Wang X., Xu X., Abassi Y.A. (2011). The xCELLigence System for Real-Time and Label-Free Monitoring of Cell Viability. Methods Mol. Biol..

[52] Kramer N., Walzl A., Unger C., Rosner M., Krupitza G., Hengstschläger M., Dolznig H. (2013). In vitro cell migration and invasion assays. Mutat. Res. Mutat. Res..

[53] Jones T.R., Kang I.H., Wheeler D.B., A Lindquist R., Papallo A., Sabatini D.M., Golland P., E Carpenter A. (2008). CellProfiler Analyst: Data exploration and analysis software for complex image-based screens. BMC Bioinform..

